# Enhanced susceptibility of triple transgenic Alzheimer’s disease (3xTg-AD) mice to acute infection

**DOI:** 10.1186/s12974-017-0826-5

**Published:** 2017-03-11

**Authors:** Rebecca Montacute, Kerry Foley, Ruth Forman, Kathryn Jane Else, Sheena Margaret Cruickshank, Stuart McRae Allan

**Affiliations:** Faculty of Biology, Medicine and Health, University of Manchester, AV Hill Building, Oxford Road, Manchester, M13 9PT UK

**Keywords:** Alzheimer’s disease, Infection, *Toxoplasma gondii*, *Trichuris muris*, Inflammation, Cytokines, Microglia, Neuroinflammation

## Abstract

**Background:**

Infection is a recognised risk factor for Alzheimer’s disease (AD) and can worsen symptoms in established disease. AD patients have higher rates of infection and are more likely to require hospital admissions due to infections than individuals without dementia. Infections have also been found to increase the risk of those over 84 years of age being diagnosed with dementia. However, few studies have investigated immune responses to infection in AD.

**Methods:**

Here, we investigated the immune responses of the triple transgenic Alzheimer’s disease (3xTg-AD) mouse model of AD to infection with the parasites *Toxoplasma gondii* and *Trichuris muris*. Cytometric bead array, histology, immunohistochemistry and immunofluorescence were used to evaluate immune responses and the effects on the brain of acute infection.

**Results:**

3xTg-AD mice, despite having comparable parasite loads, were more susceptible to infection with more severe morbidity. A worsened outcome to infection can be linked to an exaggerated immune response. 3xTg-AD mice had an increased pro-inflammatory response characterised by the production of pro-inflammatory mediators such as tumour necrosis TNF-α, IL-6, CCL5 and CXCL-1, as well as an increase in immune cell infiltration to the sites of infection. T cell responses to parasite antigen also showed elevated production of the pro-inflammatory cytokines TNF-α (10 fold) and IL-6 (twofold). We investigated whether 3xTg-AD mice had a propensity for a more Th1-dominated response using the *T. muris* worm infection and showed that akin to *T. gondii*, there was an enhanced pro-inflammatory response which was associated with retention of worms in the gut and associated pathology. Irrespective of whether the infection was one that could infect the brain or cause a local gut inflammation, 3xTg-AD mice had increased numbers of activated microglia during infection in both the cortex and the hippocampus.

**Conclusions:**

Our findings suggest that in AD, responses to infection are exaggerated outside of the CNS. Additionally, the results presented here indicate that both systemic and localised inflammation caused by an infection exacerbate neuroinflammation in AD.

**Electronic supplementary material:**

The online version of this article (doi:10.1186/s12974-017-0826-5) contains supplementary material, which is available to authorized users.

## Background

Alzheimer’s disease (AD) is a chronic neurodegenerative disease which affects over 35 million people worldwide [1]. The disease is characterised by the accumulation of Aβ protein and the deposition of tau neurofibrillary tangles (NFTs). There are no treatments to stop or reverse the effects of AD, and most patients die within 3–9 years of diagnosis [2]. Infections are a common co-morbidity in individuals with AD, with higher rates of infection and more frequent hospital admissions [3–5]. Pneumonia is the most common cause of death for patients with AD [6], and infections have been found to increase the risk of those over 84 years of age being diagnosed with dementia [7].

Over the last decade, a growing body of research has implicated inflammation in the pathology of AD. Patients taking non-steroidal anti-inflammatory drugs (NSAIDs) are significantly less likely to develop AD [8]; several genetic risk factors for AD have been identified which also have roles within the immune system, such as the myeloid cell receptor TREM2 [9], and genome-wide association studies (GWAS) suggest that there is widespread dysfunction of the immune system in AD [10]. It is currently unclear how the immune system outside of the central nervous system (CNS) is affected in AD patients or how alterations in the immune system affect the response of AD patients to an infection. Reports on differences in the immune system of AD patients are conflicting. Studies have shown increases [11] and decreases [12] in CD4+ T cells in AD patients. CD8+ T-cell numbers were increased in several studies [11–15]; however, other studies found no differences in CD8+ cells [16, 17]. Studies examining cytokine levels in AD patients are also conflicting. Increases in interleukin 6 (IL-6), IL-1β and the acute phase protein C-reactive protein have been reported in the peripheral blood of AD patients, as well as decreases in tumour necrosis factor alpha (TNF-α) [18, 19]. However, some studies have not found differences in peripheral blood and have only seen differences in AD patients following stimulation. Following lipopolysaccharide (LPS) stimulation of whole blood culture, AD patients had higher levels of IL-1β, TNF-α, IL-6 and IL-10 [11]. Dysregulation of the immune system has also been reported in the triple transgenic Alzheimer’s disease (3xTg-AD) mouse model of AD [20], including increased lymphocyte chemotaxis [21] and increased C-C chemokine receptor 6 (CCR6) expression outside of the CNS [22]. Additionally, functions of the immune system deteriorate with age, which is referred to as immunosenescence. Immunosenescence is characterised by increased susceptibility to infection and problems with lymphocyte development and function [23]. As AD is predominantly a disease of old age [2], some of the factors attributed to AD may be due to age-related immunosenescence.

It is known that systemic inflammation caused by infection can impact the brain. Brains of patients who died from sepsis have significantly increased levels of microglial activation [24], as do animals injected with lipopolysaccharide (LPS) [25]. In AD, infections worsen symptoms in patients with established disease with the rate of cognitive decline increased post infection [26, 27]. During AD, microglia are thought to proliferate and become sensitised to secondary stimuli such as a peripheral infection. This process is known as microglia priming [28]. Given the links between infection and worsened prognosis in AD, it is important to understand how systemic and localised infections affect the brain in AD. To address the immune response in AD pathology, we looked at how the immune system of a mouse model of AD responds to systemic and localised infection using two models of infection—*Toxoplasma gondii* (*T. gondii*) and *Trichuris muris (T. muris).*

Immune responses to both *T. gondii* and *T. muris* have been studied extensively in mice, so the parasites are useful tools with which to study the immune response to infection in AD. *T. gondii* and *T. muris* infect via the oral route, but *T. gondii* becomes a systemic infection and forms a lifelong chronic infection in tissues such as the eyes and central nervous system, including the brain [29]. In the brain, *T. gondii* preferentially infects areas affected by AD pathology, such as the hippocampus [30]. *T. gondii* is typified by a Th1 response. In contrast, *T. muris* remains localised to the colon and caecum, and resistance is associated with a Th2 response [31].

Previous studies have investigated chronic infection with *T. gondii* in AD mouse models. In the Tg2576 model, AD mice had higher levels of IL-10 and TFG-β in the brain 6 months post infection (PI) with the ME49 type II strain of *T. gondii,* when compared to uninfected AD mice. Infected AD mice also had reduced Aβ plaque deposition in the cortex and hippocampus and reduced learning and memory impairments in the Morris water maze and Y maze [32]. A second study investigated chronic *T. gondii* infection in the 5xFAD model of AD, also infected with the ME49 type II strain of the parasite. At 28 days PI, 5xFAD mice had reduced Aβ plaque deposits, as well as recruitment of myeloid-derived CCR2hi Ly6Chi monocytes, CCR2+ Ly6Cint, and CCR2+ Ly6Clow mononuclear cells. Both recruited cell types were shown to have an increase of Aβ phagocytosis in vivo, which may explain the decrease in Aβ plaque deposits in this study [33]. However, no study has previously investigated the effects of acute *T. gondii* infection on neuroinflammation in AD, and no studies have previously investigated infection with the parasite *T. muris* in AD mouse models.

In this study, we investigated the effects of infection with the parasites *T. gondii* and *T. muris* in the 3xTg-AD mouse model of AD. We found that 3xTg-AD mice have increased production of pro-inflammatory mediators such as TNF-α, IL-6, C-C chemokine ligand 5 (CCL5) and chemokine CXC ligand 1 (CXCL-1), as well as increased immune cell infiltration. Following *T. gondii* infection, T cell responses to parasite antigen in the spleen also showed elevated production of the pro-inflammatory cytokines TNF-α and IL-6. Irrespective of whether the infection was one that could infect the brain or cause local gut inflammation, 3xTg-AD mice had increased numbers of activated microglia during infection in both the cortex and the hippocampus.

## Methods

### Animals

3xTg-AD and background strain wild-type non-transgenic (Non-Tg) (C57BL6/129sv) male mice were bred from in house colonies, established from mice originally supplied by Frank LaFerla and Salvadore Oddo (University of California-Irvine, CA). For each parasite, we assessed specific immune responses well known to each infection model. In the *T. gondii* infection, both 5–6-month and 11–12-month-old mice were used, to investigate the effects of age on the response to infection in AD. In the *T. muris* infection, 6-month-old mice were used to allow comparisons with the responses of 5–6-month-old mice to *T. gondii* infection. Mice were housed in standard housing conditions (20 ± 2 °C, humidity 55 ± 5%, 12-h light cycle with lights on at 07:00). Mice were given ad-libitum access to food and water. All procedures were performed under appropriate personal and project licences and in accordance with the Home Office (Animals) Scientific Procedures Act (1986). Animals were housed as non-littermate controls of 3–6 animals per cage. Cages were randomly allocated into treatment groups.

### Infections

In the first set of experiments, mice were infected by oral gavage with 1 × 10^4^ tachyzoites of the type II *T. gondii* strain expressing the dimeric tomato red fluorescence protein [34]. Tachyzoites were passaged through human foreskin fibroblast cells (European collection of cell culture, Porton Down, UK) before infection. Mice were sacrificed at 5, 7 and 9 days PI. Day 5 PI was chosen to examine small intestine pathology, day 7 PI as the likely peak of systemic infection, and day 9 PI as the parasite would be likely to have reached the brain at this time point. The number of animals per group were as follows: 5–6-month-old Non-Tg mice (5 Naïve mice, 5 mice culled at day 5 PI, 7 mice at day 7 PI and 6 mice at day 9 PI), 5–6-month-old 3xTg-AD mice (5 Naïve mice, 5 mice culled at day 5 PI, 7 at day 7 PI and 4 at day 9 PI), 11–12-month-old Non-Tg mice (6 Naïve mice, 5 culled at day 7 PI and 4 at day 9 PI) and 11–12-month-old 3xTg-AD mice (3 Naïve, 4 culled at day 7 PI and 3 at day 9 PI). In a second experiment, 10-month-old mice were infected by intraperitoneal (IP) injection with 1 × 10^4^ tachyzoites of the same strain of *T. gondii*. The Y maze behavioural test was carried out at 5 days PI; the open field behavioural test was carried out at 6 days PI and culled at day 9 PI. In both experiments, animals were culled using a rising concentration of carbon dioxide (CO_2_). All animals were infected in the morning between 9 and 11 a.m. on day 0 PI. Mice were weighed and checked daily for signs of ill health throughout infection. No animals lost more than 10% of their original body weight during the infection.

In the third set of experiments, mice were infected with approximately 40 viable eggs of the E strain of *T. muris* by oral gavage and killed at 35 days PI. Maintenance of the *T. muris* life cycle and production of excretory/secretory (E/S) antigen were carried out as described previously [35]. Mice which are resistant to *T. muris* typically expel the parasite between 11–28 days PI. In susceptible mice, which are unable to expel the parasite, *T. muris* worms can usually be detected from 35 days PI. Each experimental group contained 4 animals, and all animals were 6 months of age. Animals were culled using a rising concentration of CO_2_. Immediately post-mortem, the caecum was cut and washed out with water to flush out the faecal contents, and the numbers of *T. muris* worms were counted at ×4 magnification using a dissection microscope. Mice were weighed daily and checked daily for signs of ill health for the first 2 weeks of infection, after which animals were checked every few days. No animals lost more than 10% of their original body weight during the infection.

### Splenocyte and MLN cell culture and cytokine analysis

Spleens were taken at autopsy and strained through a 100-μm cell strainer (Fisher Scientific, Loughborough, UK), and red blood cells were removed by treatment with a red blood cell lysis buffer (Sigma Aldrich, MO, USA). Cells from *T. gondii*-infected mice were plated at 1 × 10^6^ cells/well and incubated in the presence of STAg (soluble tachyzoite antigen) (25 μg/ml) for 24 h or ConA (2.5 μg/ml) for 72 h. STAg was produced from cultured tachyzoites, freeze thawed in liquid nitrogen and filtered.

Single-cell suspensions were prepared from mesenteric lymph nodes (MLNs) from *T. muris*-infected mice and plated at 1 × 10^6^ cells/well of a 96-well plate. Cells were incubated with E/S antigen (50 μg/ml) for 48 h or ConA (2.5 μg/ml) for 72 h. All cells were incubated at 37 °C, 5% CO_2_ and after incubation supernatants were harvested and stored at −80 °C. Levels of IL-10, 1 L-13, IL-4, IL-6, IL-1β, IL-1α, interferon gamma (IFN-γ), TNF-α, CXCL-1, CCL5 and CCL2 were determined using a custom cytometric bead array (CBA) according to the manufacturer’s instructions (BD Biosciences, Oxford, UK), and plates were read using a MACSQuant Analyser (Miltenyi Biotec, Cologne, Germany). Analysis was carried out using FCAP Array™ Software (BD Biosciences).

### Histology

Liver and small intestine samples from *T. gondii*-infected mice and colon samples from *T. muris*-infected mice were fixed in neutral-buffered formalin for 24 h, processed and embedded in paraffin wax. Five-μm sections were dewaxed, rehydrated and stained using a standard haematoxylin and eosin staining method. In liver samples, inflammatory foci were counted; an average was taken for each mouse of 3 fields of view at 10× magnification. In small intestine samples, the length of 20 villi were measured and averaged per mouse at 10× magnification. Liver and small intestine measurements were carried out using Pannoramic Viewer software (3D HISTECH, Budapest, Hungary). In colon samples, the length of 20 crypts was measured using ImageJ (National Institutes of Health, MD, USA) and averaged per mouse at 10× magnification. All slides were measured and counted blind in a randomised order.

### Immunohistochemistry

Colon samples from *T. muris*-infected mice were flash frozen in optimal cutting temperature compound (OCT; Thermo Fisher Scientific, Runcorn, UK) and stored at −80 °C. Five-μm sections were cut using a cryostat (Leica Biosystems, Milton Keynes, UK). Sections were incubated with primary antibodies to CD45 5 μg/ml (Bio-Rad, Langford, UK) or F4/80 5 μg/ml (Bio-Rad, Langford, UK). Slides were incubated with avidin and biotinylated horseradish peroxidase macromolecular complex kit (ABC; Vector Laboratories, Peterborough, UK), then developed using 3,3’-diaminobenzidine (DAB; Vector Laboratories, Peterborough, UK). Slides were then washed and counterstained with HaemQS (Vector Laboratories, Peterborough, UK). Slides were imaged using an Olympus BX51 upright microscope using ×10 objective and captured using a Coolsnap ES camera (Photometrics, Tucson, USA) through MetaVue Software (Molecular Devices, Sunnyvale, USA). CD45+ or F4/80+ cells were counted for 20 crypts from three disparate areas in the colon of each mouse and then averaged. All slides were measured and counted blind in a randomised order.

### Immunofluorescence

Immunohistochemistry was performed on free-floating brain sections. Non-specific binding was blocked by treatment with 2% normal donkey serum (NDS) (Vector Laboratories, Peterborough, UK) for 1 h at room temperature. Sections were incubated overnight in antibody diluent (0.1 M PBS, 0.1% Triton, 2% NDS) using the following primary antibodies: rabbit anti-mouse Iba1 2 μg/ml (Wako Chemicals, Düsseldorf, Germany) and goat anti-mouse Ds-Red 1 μg/ml (Stratech, Newmarket, UK). Antigens were visualised with the appropriate (Alexa Fluor 488 1 μg/ml Molecular Probes or Alex Fluor 594 1 μg/ml Santa Cruz Biotechnology, Santa Cruz, USA) secondary donkey anti-sera for 2 h at room temperature. Sections were mounted onto gelatin-coated slides, dehydrated and coverslipped using ProLong Gold Antifade reagent (Molecular Probes, Thermo Fisher Scientific, Runcorn, UK). Images were collected using an Olympus BX51 upright microscope using ×10 objective and captured using a Coolsnap ES camera (Photometrics, Tucson, USA) through MetaVue Software (Molecular Devices, Sunnyvale, USA). Specific band pass filters for DAPI, FITC and Texas red were used to prevent bleed through from one channel to the next. Images were processed and analysed using ImageJ (National Institutes of Health, MD, USA).

Activated microglia were identified as showing (1) increased Iba1 immunopositivity, (2) enlarged and/or amoeboid cell body and (3) complete or partial loss of thin, elongated processes. Round-shaped, small Iba1-positive cells with leucocyte morphology were not counted. Activated microglia were counted through the cortex and the hippocampus. In the cortex for each animal, images were taken from the top, middle and bottom of the cortex from three brain sections from the front, middle and back of the brain. In the hippocampus, averages were taken from CA1, CA2, CA3 and the dentate gyrus (DG) of sections from the front, middle and back of the brain. All slides were measured and counted blind in a randomised order.

### Statistics

All data analysis was carried out on blinded and randomised samples. Power calculations were carried out using the Statistical Solutions Power and Sample Size Calculator (Statistical Solutions, WI, USA) using previous data from our group and calculated for an effect size of 80%. All statistical analyses were carried out using GraphPad Prism version 6.04 (GraphPad Software, San Diego, USA). For two groups, parametric data were analysed using Student’s *t* test, and for data with unequal variances, Welch’s correction was applied. All other statistical analyses were performed using two-way analysis of variance with Tukey’s multiple comparison test. All data are expressed as mean ± standard error of the mean (SEM). *P* values of <0.05 were considered significant.

## Results

### Increased inflammation in 3xTg-AD mice following infection with *T. gondii*

**Fig. 1:**
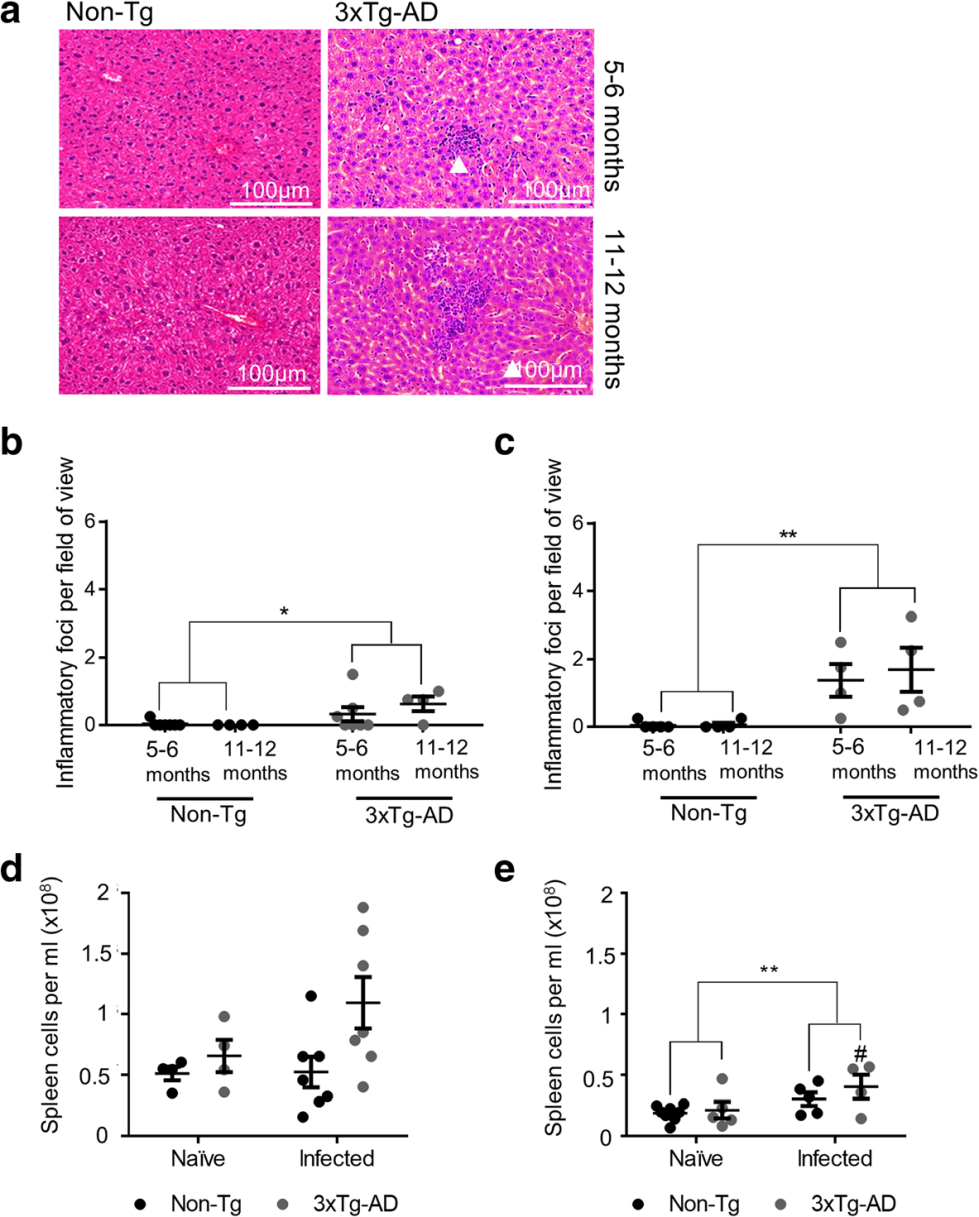
Inflammatory cell infiltration following *T. gondii* infection in 3xTg-AD mice. Mice were infected with 1 × 10^4^ tachyzoites of *T. gondii* by oral gavage. **a** Representative images of inflammatory foci infiltration in the livers of Non-Tg and 3xTg-AD mice post infection. Numbers of inflammatory foci in the liver were analysed at day 7 (**b)** and day 9 (**c)** post infection. Splenocyte numbers were counted in **d** 5–6-month-old mice and **e** 11–12-month-old mice at 7 days post infection. *P ≤ 0.05; **P ≤ 0.01. All data are shown as mean ± SEM

In the liver, numbers of inflammatory foci were significantly higher in infected 3xTg-AD mice than in infected Non-Tg mice at both 7 days (Fig. 1b; *P* ≤ 0.05) and 9 days (Fig. 1c; *P* ≤ 0.01) PI. Significant increases in inflammatory foci were seen in both 5–6-month-old and 11–12-month-old 3xTg-AD mice compared with controls. No inflammatory foci were seen in either Non-Tg or 3xTg-AD Naïve mice at either age (data not shown). *T. gondii* infection is also associated with a transient ileitis in the small intestine, another major site of acute infection. Although we saw evidence of ileitis post infection, there was no difference in the response between 3xTg-AD and Non-Tg mice (data not shown).

The immune response to *T. gondii* infection is associated with splenocyte cell proliferation as one of the sites of immune activation. Splenocyte numbers were not seen to be significantly different between infected 5–6-month-old Non-Tg mice and 3xTg-AD mice (Fig. 1d), but in 11–12-month-old mice, spleen cell numbers were significantly increased (*P* ≤ 0.05) in infected 3xTg-AD mice compared to naïve Non-Tg mice (Fig. 1e).

### 3xTg-AD mice have an increased pro-inflammatory response following infection with *T. gondii*

**Fig. 2:**
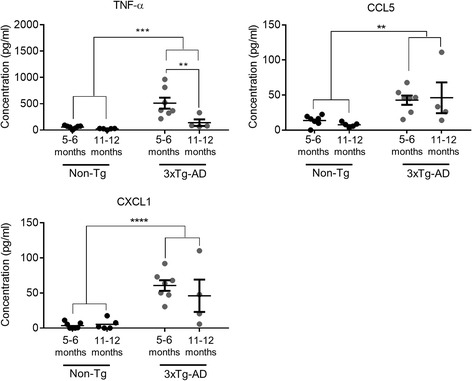
Cytokine and chemokine expression after STAg stimulation of splenocytes following infection with *T. gondii.* Splenocytes from day 7 PI were isolated and stimulated with STAg (25 μg/ml) for 24 h, and cytokine and chemokine levels were measured using a CBA. **P ≤ 0.01; ***P ≤ 0.001; ****P ≤ 0.0001. All data are shown as mean ± SEM

We next wanted to determine whether the increase in immune cells seen in 3xTg-AD mice following *T. gondii* infection was also associated with increased cytokine and chemokine secretion. Infection with *T. gondii* triggers a rapid Th1 cell-mediated response, which peaks at around day 7 PI. Parasite specific responses were analysed by stimulating splenocytes at 7 days PI with STAg. Significantly increased levels of the cytokine TNF-α (*P* ≤ 0.001) and the chemokines CCL5 (*P* ≤ 0.01) and CXCL-1 (*P* ≤ 0.0001) were found in infected 3xTg-AD mice compared to infected Non-Tg mice. This trend for elevated levels of pro-inflammatory cytokines following STAg stimulation was consistent across two additional independent *T. gondii* infection time courses we carried out in 3xTg-AD mice (data not shown). Interestingly, TNF-α levels were significantly lower in infected 11–12-month-old 3xTg-AD mice than in infected 5–6-month-old 3xTg-AD mice (*P* ≤ 0.01) (Fig. 2). STAg stimulation was also carried out at day 9 PI; however, only low levels of cytokine expression were observed at this time point, and no differences were seen between 3xTg-AD and Non-Tg mice (data not shown).

### Increased microglial activation in 3xTg-AD mice following infection with *T. gondii*

**Fig. 3:**
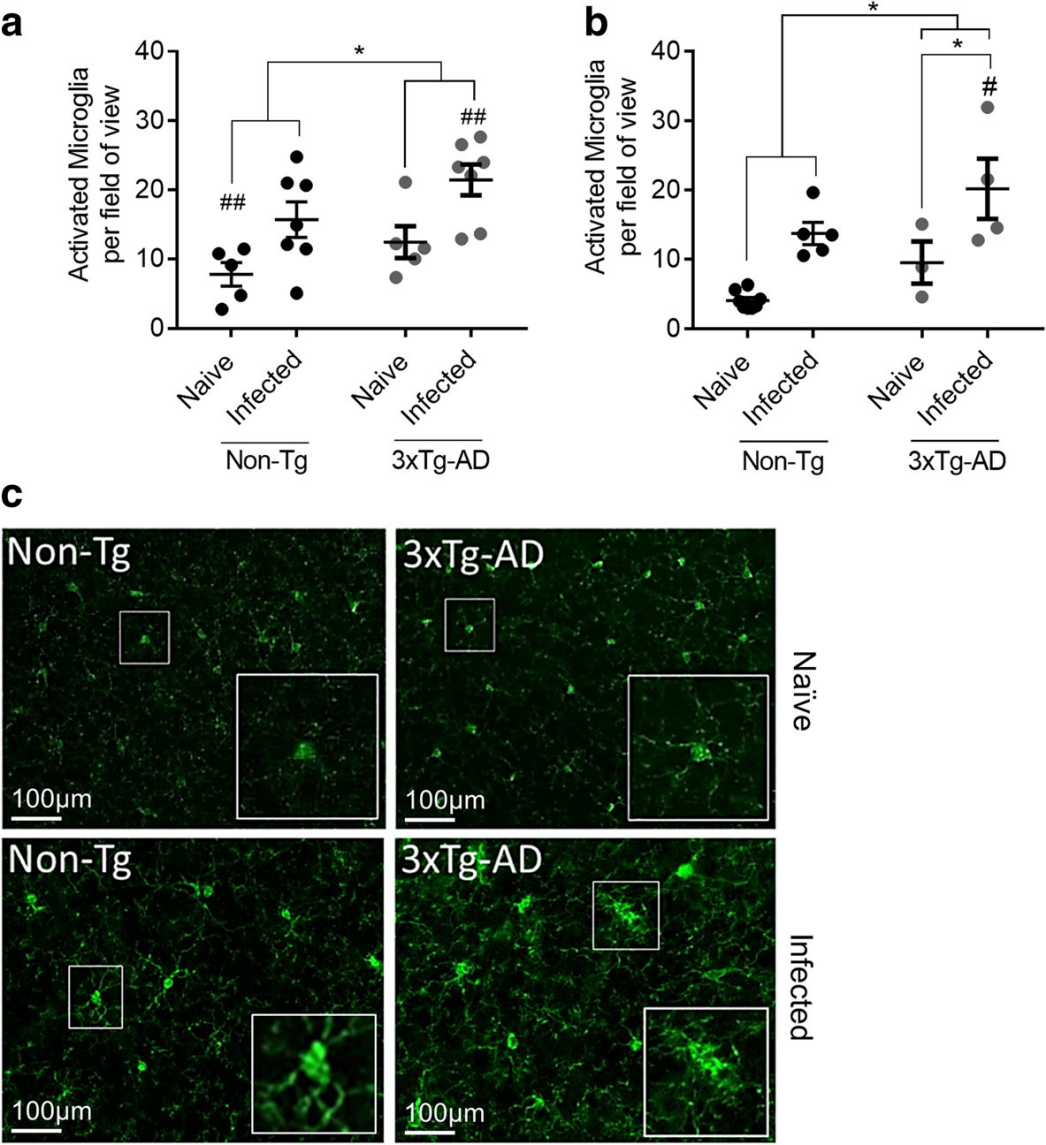
Microglial activation following *T. gondii* infection in 3xTg-AD mice 7 days PI. Activated microglia were identified by increased Iba1 positivity, thickened processes and irregular cell bodies. Microglial activation is shown in the cortex of **a** 5–6-month-old mice and **b** 11–12-month-old mice at day 7 PI. **c** Iba1 staining in the cortex of naïve and infected mice at day 7 PI; *insets* show representative images from each group. *Scale bars* show 100 μm. *P < 0.05; **P ≤ 0.01; ***P ≤ 0.001. Secondary comparisons are indicated by ^##^ *P* ≤ 0.01 above groups which differ significantly from one another. All data are shown as mean ± SEM

The possibility that elevated serum and splenic cytokine responses would be associated with greater microglial activation in the brain was explored using immunohistochemistry. Iba1 staining was used to identify activated microglia, although it may also have detected some local or infiltrating macrophages. At day 7 PI, which corresponds to the time when it was unlikely for parasite to be present in the brain, infected 5–6-month-old 3xTg-AD mice had significantly more activated microglia in the cortex than naïve Non-Tg mice (*P* ≤ 0.01; Fig. 3a). At 11–12 months of age at day 7 PI, infected 3xTg-AD also had significantly higher numbers of activated microglia in the cortex compared with Non-Tg mice (*P* ≤ 0.05; Fig. 3b).

**Fig. 4:**
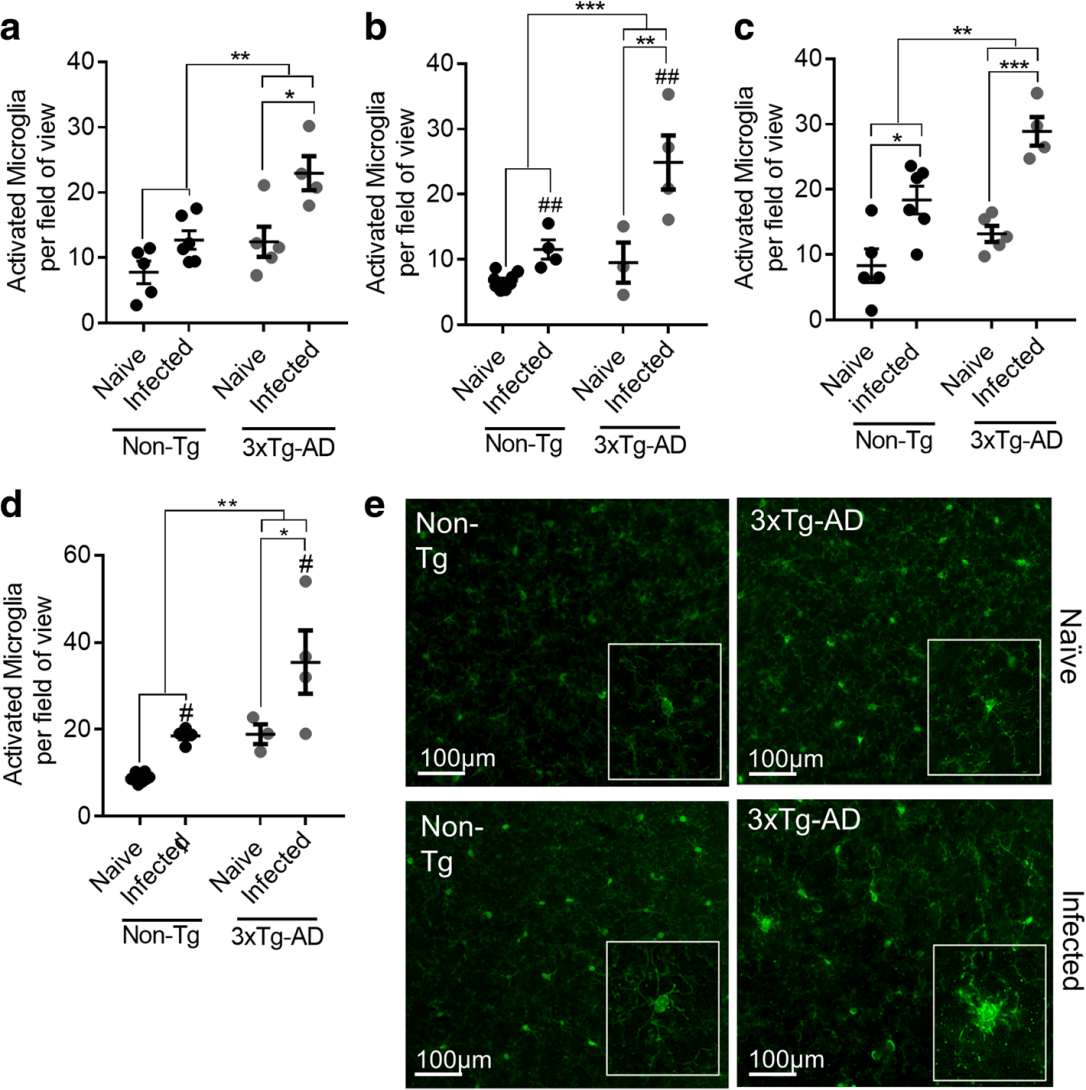
Increased microglial activation following *T. gondii* infection in 3xTg-AD mice. Activated microglia were identified by increased Iba1 positivity, thickened processes and irregular cell bodies. Microglial activation is shown in the cortex of 5–6-month-old **a** and 11–12-month-old **b** mice at day 9 PI, and the hippocampus of 5–6-month-old **c** and 11–12-month-old **d** mice at day 9 PI. **e** Iba1 staining in the cortex of naïve and infected mice at day 9 PI; *insets* show representative images from each group. *Scale bars* show 100 μm. *P < 0.05; **P ≤ 0.01; ***P ≤ 0.001. Secondary comparisons are indicated by ^#^ *P* < 0.05 or ^##^ *P* ≤ 0.01 above groups which differ significantly from one another. All data are shown as mean ± SEM

At day 9 PI, a time point in which there should be parasite present in the brain, microglial activation increased significantly in infected 3xTg-AD mice in both the cortex (*P* ≤ 0.05) (Fig. 4a) and the hippocampus (*P* ≤ 0.001) (Fig. 4c) of in 5–6-month-old mice. Infected Non-Tg mice had increased microglial activation in the hippocampus (*P* ≤ 0.05) (Fig. 4c) but not in the cortex (Fig. 4a), and the increase in the hippocampus was smaller than that seen in 3xTg-AD mice. At 11–12 months of age, microglial activation was significantly higher in infected 3xTg-AD mice compared to non-infected 3xTg-AD mice in both the cortex (*P* ≤ 0.01) and the hippocampus (*P* ≤ 0.05). In contrast, at 11–12 months, no significant increase was seen in the numbers of activated microglia in infected Non-Tg compared to non-infected Non-Tg mice (Fig. 4b, d).

**Fig. 5:**
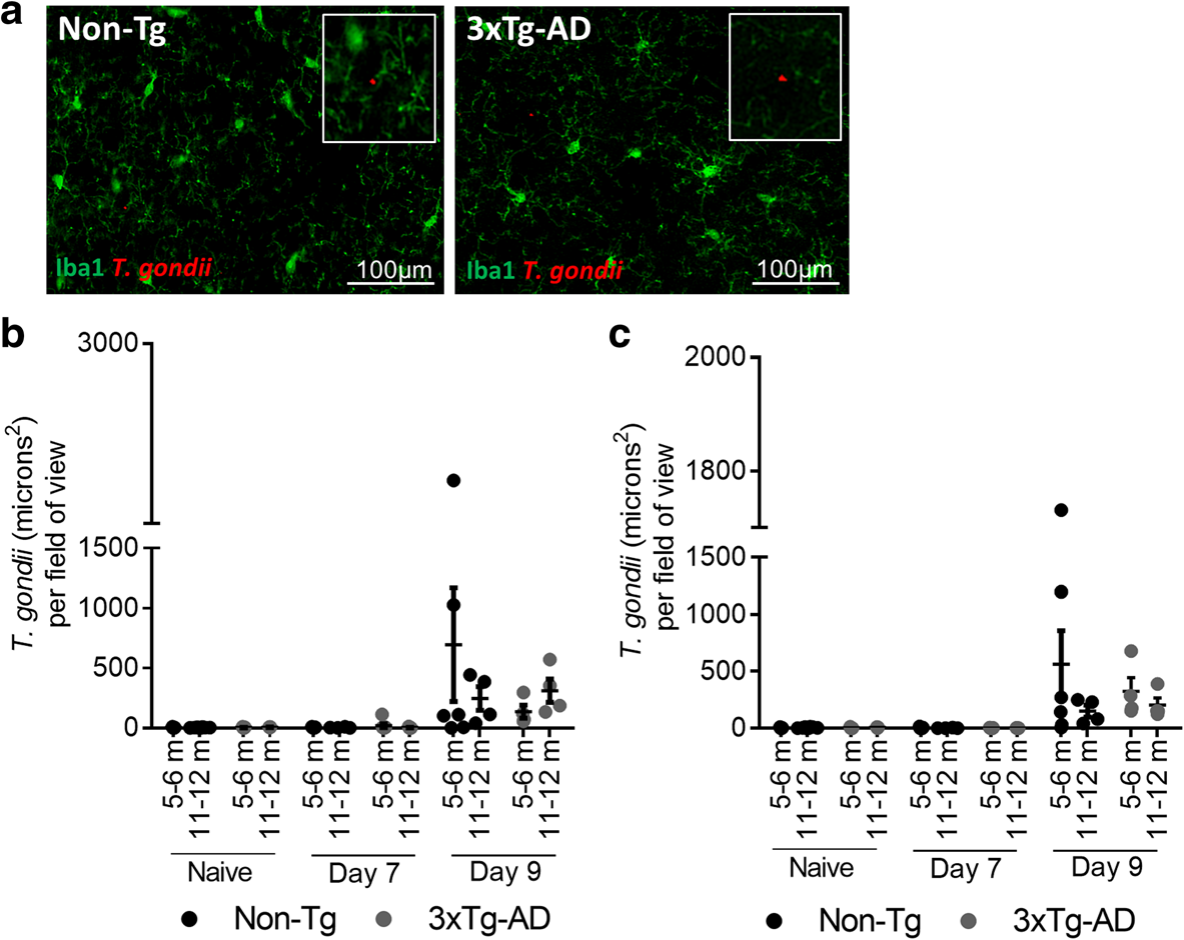
Parasite burden in the brain following *T. gondii* infection. **a** Representative images of *T. gondii* and Iba1 staining in the cortex of Non-Tg and 3xTg-AD mice at day 9 PI. Parasite burden in the cortex in infected mice in the **b** cortex and the **c** hippocampus. All data are shown as mean ± SEM

The possibility that microglial activation correlated with parasite burden or presence of parasite in microglial areas was investigated. At day 7, only very low levels of parasites were detected in the brains of infected mice, and the numbers of parasites detected in the brain increased at day 9 PI (for qPCR see also Additional file 1: Figure S1). There was no significant difference in the amount of *T. gondii* parasites in the brains of 3xTg-AD to Non-Tg mice at day 7 or day 9 PI in either the cortex (Fig. 5b) or the hippocampus (Fig. 5c). Naïve mice had no *T. gondii* parasites in the brain. Parasites at day 9 were not found to be localised to activated microglia. This data suggests that the enhanced microglial activation is not due to increased parasite load in the brains of 3xTg mice. The possibility that the microglial activation was due to enhanced local brain cytokines was next investigated by qPCR on the brains 9 days PI with *T. gondii*. No significant differences in the expression of IL-1α, IL-1β or TNF-α were seen in the brains of infected 3xTg-AD mice compared to naïve 3xTg-AD, naïve Non-Tg or infected Non-Tg mice. Levels of IFN-γ and IL-12 were too low to detect (see Additional file 2: Figure S2). The possibility that infection could impact behaviour was also assessed. No significant differences were found between infected Non-Tg and infected 3xTg-AD animals in behavioural tests (see Additional file 3: Figure S3).

### Elevated pro-inflammatory responses in 3xTg-AD mice after *T. muris* infection

**Fig. 6:**
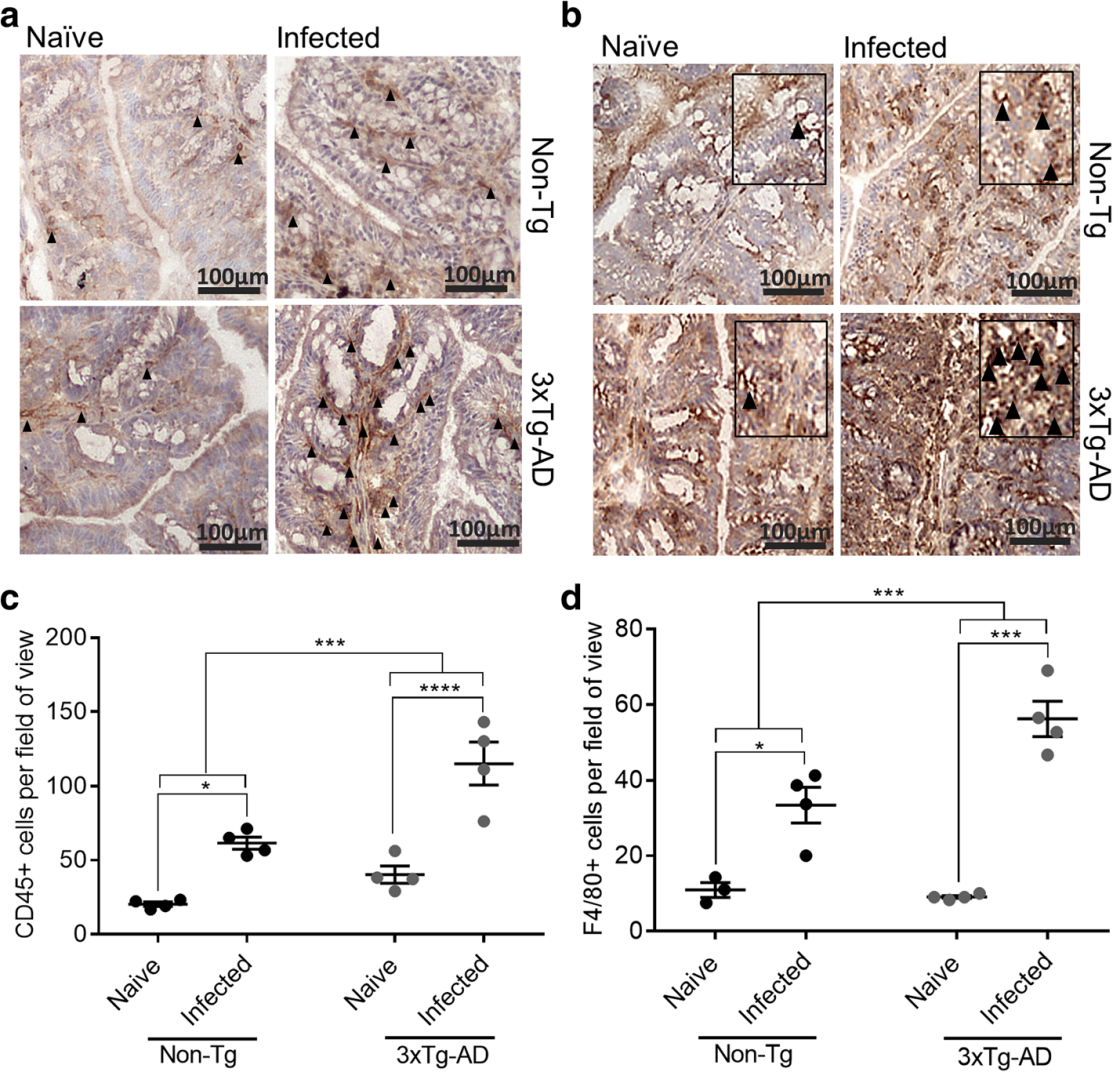
Exaggerated inflammatory cell responses following infection with *T. muris* in 3xTg-AD mice*.* At 35 days PI colon sections were stained for **a** CD45 and **b** F4/80 and the average 20 crypts per area counted over three disparate areas for **c** CD45 and **d** F4/80. Images show representative staining in 3xTg-AD and Non-Tg mice. *P < 0.05; **P ≤ 0.01; ***P ≤ 0.001. All data are shown as mean ± SEM

3xTg-AD mice were infected with *T. muris* to investigate whether exaggerated immune responses were also seen in a localised infection that does not infect the brain. During acute *T. muris* infection, the major site of infection and inflammatory cell infiltration is the colon, and thus, we determined numbers of CD45 positive cells in the colon. CD45^+^ cell levels were increased following infection, with a larger increase in 3xTg-AD mice (*P* ≤ 0.0001) compared to Non-Tg animals (*P* ≤ 0.05) (Fig. 6a, c). We also measured the number of F4/80^+^ macrophages in the colon following infection with *T. muris*. Significantly higher numbers of macrophages were seen in both Non-Tg and 3xTg-AD mice following infection; however, the increase following infection was larger in 3xTg-AD mice than in Non-Tg mice, with a significant interaction effect seen between genotype and infection (*P* ≤ 0.01) (Fig. 6b, d). *T. muris* infection is associated with altered crypt architecture in the colon; however, we saw no differences in crypt length between infected Non-Tg and 3xTg-AD mice (data not shown).

**Fig. 7:**
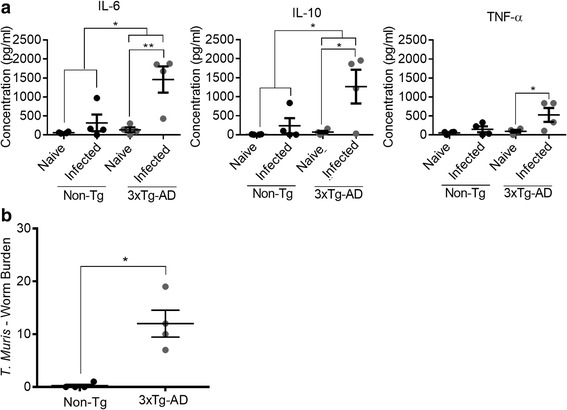
Responses in 3xTg-AD mice to infection with the nematode parasite *T. muris.* **a** 6-month-old mice were infected with 40 viable eggs of the E strain of *T. muris* (low dose) and sacrificed at 35 days PI. MLNs from *T. muris*-infected mice were plated 1 × 10^6^ cells/well of a 96-well plate and stimulated with E/S antigen (50 μg/ml) for 48 h; cytokine and chemokine levels were measured by CBA. **b** *T. muris* worm burden was enumerated. *Scale bars* show 100 μm. *P < 0.05; **P ≤ 0.01; ***P ≤ 0.001. All data are shown as mean ± SEM

To determine the specific response to *T. muris*, MLNs were stimulated with the *T. muris* specific antigen E/S. Following E/S stimulation, infected 3xTg-AD mice produced significantly larger amounts of the cytokines IL-6 (*P* ≤ 0.01), IL-10 (*P* ≤ 0.05) and TNF-α (*P* ≤ 0.01) than naïve 3xTg-AD mice and significantly larger amounts of IL-6 (*P* ≤ 0.05) and IL-10 (*P* ≤ 0.05) than infected Non-Tg mice. No differences were seen between naïve Non-Tg and infected Non-Tg mice following E/S stimulation of MLNs (Fig. 7a). Non-Tg mice were able to expel the parasite, with the exception of one mouse that had a single worm remaining in the colon. 3xTg-AD mice were unable to expel the parasite and still had worms in their gut at day 35 suggesting they had developed a chronic infection (*P* ≤ 0.01; Fig. 7b).

**Fig. 8:**
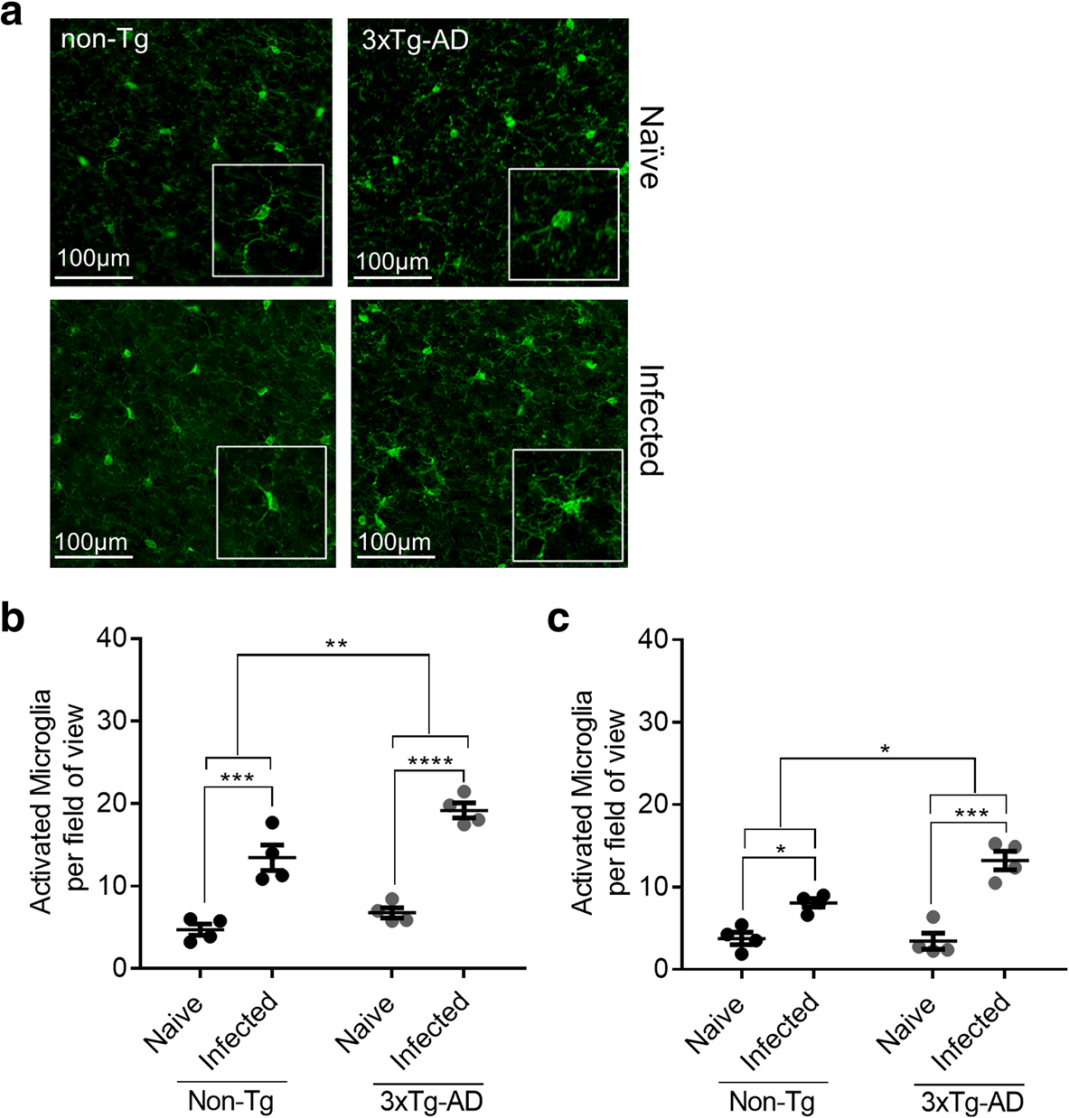
Increased microglial activation following *T. muris* infection in 3xTg-AD mice. **a** Brains were stained with Iba1, and activated microglia were identified. Images show representative staining in the cortex of 3xTg-AD and Non-Tg mice. Enumeration of activated microglia in **b** the cortex and **c** the hippocampus of 6-month-old mice following infection with *T. muris*

Microglial activation was also assessed following infection with *T. muris*; significant increases in activated microglia were seen in both 3xTg-AD mice and Non-Tg mice in both the cortex (*P* ≤ 0.01) (Fig. 8b) and the hippocampus (*P* ≤ 0.05) (Fig. 8c) relative to naïve animals following infection. The increase in microglial activation following infection was greater in 3xTg-AD mice than in Non-Tg mice, in the cortex (*P* ≤ 0.01) and the hippocampus (*P* ≤ 0.01).

## Discussion

Infection is a common co-morbidity in AD, which is thought to impact the quality and length of life of AD patients. AD patients are more likely to get an infection [36], have worsened symptoms from infection and more often need hospitalisation from conditions such as pneumonia [5, 37]. This study is the first to show that in an AD mouse model, regardless of whether the infection reaches the brain, the immune system’s response to an infection is altered outside of the brain. Outside of the brain, 3xTg-AD mice had increased immune cell infiltration and higher levels of pro-inflammatory cytokine and chemokine production following infection. Additionally, this study demonstrated that in 3xTg-AD mice, a local distant infection is also linked to higher levels of inflammation in the brain, with increased microglial activation following infection. There are not currently any drugs available which can stop or reverse the effects of AD. However, if infections are worsening AD outcome, better prevention and/or treatment of infection could improve the quality of life of AD patients.

This study demonstrated a generalised pro-inflammatory response in 3xTg-AD mice to infection, resulting in worsened pathology and altered immunity. Consistent with our findings, previous work has found several differences in the immune systems of 3xTg-AD mice compared to Non-Tg animals. Naïve 3xTg-AD mice have been previously shown to have abnormal enlargement of the spleen and liver, an increased number of double-negative CD4^−^CD8^−^ splenocytes [20], increased lymphocyte chemotaxis [21] and increased C-C chemokine receptor 6 (CCR6) expression outside of the CNS [22]. It is not clear how altered immune responses to infection outside of the CNS in AD mice relate to immune responses in AD patients although differences in the immune system are known to occur in AD patients. Studies in human AD patients have seen increased levels of TNF-α in serum from patients with severe AD [12]. AD patients are more likely to be hospitalised for infections including pneumonia [3], gastroenteritis [4] and urinary tract infections (UTIs) [5] than the general population, which may be due to exaggerated immune responses to infection. Additionally, following lipopolysaccharide (LPS) stimulation of whole blood from AD patients, higher levels of IL-1β, TNF, IL-6, and IL-10 have been reported [11]. AD patients with periodontitis have higher levels of serum IL-6 and TNF-α than uninfected controls [38]. The elevated pro-inflammatory responses seen in AD patients are similar to our observations in 3xTg-AD mice, suggesting that our findings are likely to be relevant to AD patients.

Previous work in 3xTg-AD mice has found elevated levels of TNF-α in the entorhinal cortex of AD mice [39], but we found no change in the levels of any of the cytokines measured that were found in infected or naive mice. However, in this study, cytokine levels were measured in the whole brain; therefore, any localised changes would not have been detected. In infection with the parasite *T. gondii,* microglial activation occurred from day 7 PI when we saw only very low levels of parasite in the brain; it is likely to be due to inflammation outside of the CNS. Furthermore, microglial activation was observed following *T. muris* infection, a parasite model which does not infect the brain and causes a localised infection in the large intestine. Microglia are thought to be primed by ongoing pathology in the brain during AD and to be activated following immune challenge outside of the CNS [28]. In 3xTg-AD mice, infection with the mouse hepatitis virus by intracranial injection increased levels of microglial activation and infiltration of macrophages and T cells. In keeping with our findings, peripheral administration of LPS has been shown to result in microglial activation in the 3xTg-AD mouse model [40]. However, LPS administration is a simplified model for an inflammatory stimulus as it will trigger a restricted repertoire of responses. Additionally, the doses of LPS administered to mice are much higher than doses which, in humans, would cause septic shock, so the use of LPS is unlikely to be a good model for the impacts of a systemic infection on human disease [41]. Here, we have shown that microglial activation occurs during common and well-characterised infections. Our findings are similar to observations in patients. AD patients who have suffered from an infection also have a greater rate of cognitive decline than patients without infection, a difference linked to increased levels of serum IL-1β [26]. A decrease in cognitive decline in AD patients following systemic inflammation has been seen to last at least 6 months after the event and has also been linked to higher serum levels of TNF [27].

*T. gondii* is a relevant infection in AD patients as it is a common infection in developed countries where AD is most common, with infection rates as high as 80% in countries such as France and Germany and rates of 10–20% in the USA although due to the lack of screening for *T. gondii* in the UK and USA, it is likely that infection rates will be higher [42]. Increased seroprevalance of *T. gondii* infection was found in individuals with AD, but whether this represents a risk factor for AD development or is associated with changes in pathology has not been determined [43]. In mouse studies, chronic infection with *T. gondii* has been suggested to be beneficial with reduced plaque burden and higher levels of anti-inflammatory cytokines [32]. *T. gondii* is a lifelong infection but can be acquired for the first time at any stage in life. In our study, the 3xTg-AD mice were unusually susceptible to acute infection, and thus, we could not investigate effects of chronic infection, but notably, several of the findings in acute infection reflect results found following chronic infection in AD. Previous work on chronic *T. gondii* infection in the Tg2576 AD mouse model found that levels of the pro-inflammatory cytokine IFN-γ remained unchanged in the brain following infection [32]. Similarly, we reported unchanged levels of the pro-inflammatory cytokines IL-1α, IL-1β and TNF-α in the brain following acute infection with *T. gondii* even at time points correlating with parasite presence in the brain. Additionally, following chronic *T. gondii* infection in the 5xFAD mouse model, increased Iba1 positive staining was noted in infected mice [33] and also we saw increased microglial activation acute infection.

Functions of the immune system are known to deteriorate with age, which is referred to as immunosenescence. AD is predominantly a disease of old age [2]; therefore, some of the factors attributed to AD may be due to age-related immunosenescence. No previous studies in animals or humans have investigated how immune responses in 3xTg-AD mice alter with age. Our findings suggest that some specific aspects of the immune response may be dampened with age in these animals, as levels of TNF-α in 3xTg-AD mice following infection were significantly lower in 11–12-month-old mice compared to 5–6-month-old animals, although still higher than in WT animals of the same age. However, increased immune cell infiltration was seen in AD mice irrespective of age. Our data therefore suggest that despite increased age, there is not widespread immunosenescence in older mice. It may be that in mice older than the 11–12-month-old animals used in this study, we see this effect drop off, and this might be interesting to be addressed in future studies.

## Conclusions

The data presented here suggests that in AD, responses to infection are exaggerated outside of the CNS and that both systemic and localised inflammation caused by an infection increases neuroinflammation in AD and thus have implications for the management of AD.

## Additional files

Additional file 1: Figure S1. 11-month-old mice were infected with 1 × 10^4^ tachyzoites of the PRU strain of *T. gondii* by oral gavage and culled at day 9 PI. One hemisphere of the brain was homogenised, RNA extracted and qPCR carried out for SAG-1 (tachyzoites) and Cysts. Data analysed by two-way ANOVA with Tukey’s multiple comparisons test. All data are shown as mean ± SEM. (TIF 17 kb)
Additional file 2: Figure S2. Cytokine levels in the brain at day 9 PI with *T. gondii* in 3xTg-AD mice. Mice were infected with 1 × 10^4^ tachyzoites of *T. gondii* by oral gavage. One hemisphere of the brain was homogenised, RNA extracted and qPCR carried out on (A) 5–6-month-old and (B) 11–12-month-old animals. Data analysed by two-way ANOVA with Tukey’s multiple comparison test. All data are shown as mean ± SEM. (TIF 101 kb)
Additional file 3: Figure S3. 10-month-old 3xTg-AD mice were infected with 1 × 10^4^ tachyzoites of *T. gondii* by IP injection. Open field behavioural testing was carried out 6 days PI. During open field, the (A) number of line crossings, (B) time spent mobile, (C) number of rears and (D) number of defecations during the test were measured. The Y-maze behavioural test was carried out 5 days PI. (E) Percentage alternations and (F) the number of arm entries in the Y-maze were measured. Data analysed by two-way ANOVA with Tukey’s multiple comparisons test, **p* < 0.05; **p < 0.01. All data are shown as mean ± SEM. (TIF 133 kb)

## Abbreviations

AD: Alzheimer’s disease
CBA: Cytometric bead array
CCL: C-C chemokine ligand
CCR: C-C chemokine receptor
CNS: Central nervous system
CO_2_: Carbon dioxide
CXCL: Chemokine CXC ligand
DG: Dentate gyrus
GWAS: Genome-wide association studies
IFN-γ: Interferon gamma
IL: Interleukin
LPS: Lipopolysaccharide
MLNs: Mesenteric lymph nodes
NDS: Normal donkey serum
NFTs: Tau neurofibrillary tangles
NSAIDs: Non-steroidal anti-inflammatory drugs
OCT: Optimal cutting temperature
SEM: Standard error of the mean
TNF-α: Tumour necrosis factor alpha
UTIs: Urinary tract infections
