# Supplementary figures and images for: Enhanced susceptibility of triple transgenic Alzheimer’s disease (3xTg-AD) mice to acute infection
Source: J Neuroinflammation. 2017 Mar 11;14:50. doi: 10.1186/s12974-017-0826-5 (PMC5346250; doi:10.1186/s12974-017-0826-5)

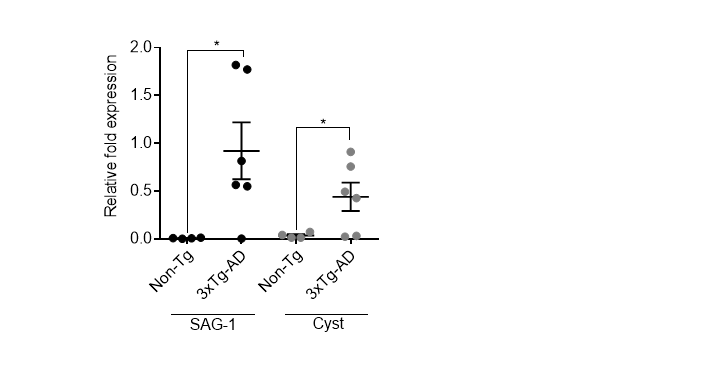

Supplement: Additional file 1: Figure S1. — 11-month-old mice were infected with 1 × 10^4^ tachyzoites of the PRU strain of T. gondii by oral gavage and culled at day 9 PI. One hemisphere of the brain was homogenised, RNA extracted and qPCR carried out for SAG-1 (tachyzoites) and Cysts. Data analysed by two-way ANOVA with Tukey’s multiple comparisons test. All data are shown as mean ± SEM. (TIF 17 kb) [file 12974_2017_826_MOESM1_ESM.tif]

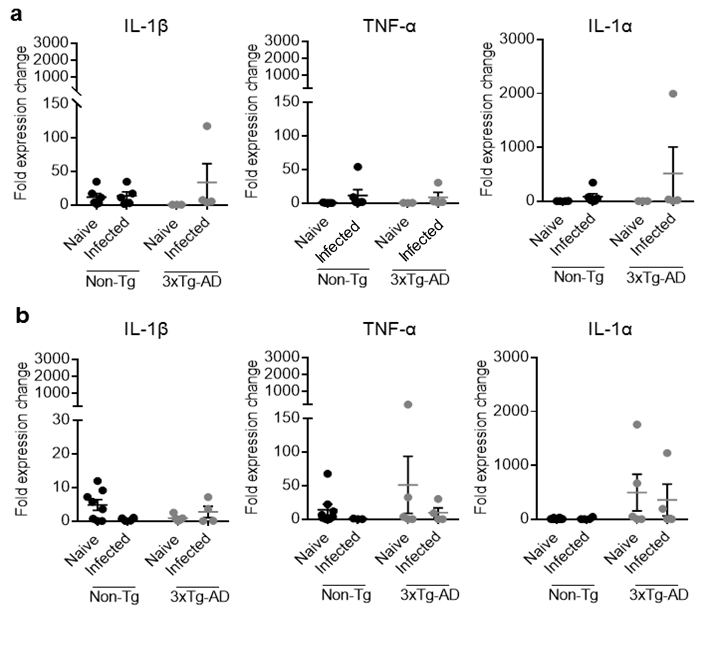

Supplement: Additional file 2: Figure S2. — Cytokine levels in the brain at day 9 PI with T. gondii in 3xTg-AD mice. Mice were infected with 1 × 10^4^ tachyzoites of T. gondii by oral gavage. One hemisphere of the brain was homogenised, RNA extracted and qPCR carried out on (A) 5–6-month-old and (B) 11–12-month-old animals. Data analysed by two-way ANOVA with Tukey’s multiple comparison test. All data are shown as mean ± SEM. (TIF 101 kb) [file 12974_2017_826_MOESM2_ESM.tif]

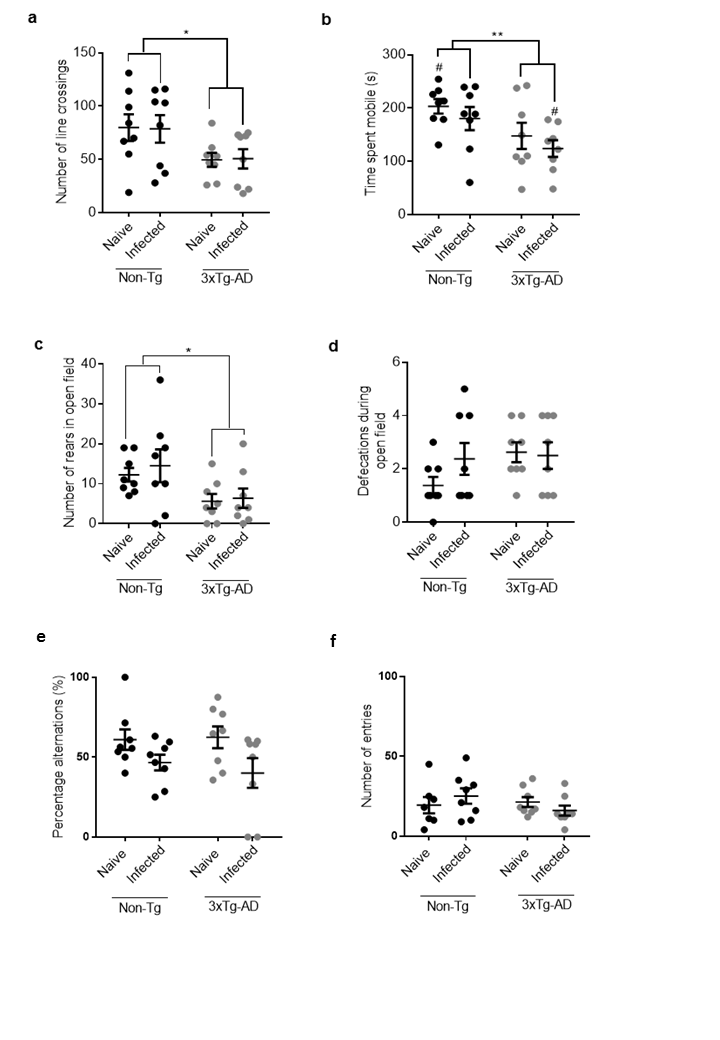

Supplement: Additional file 3: Figure S3. — 10-month-old 3xTg-AD mice were infected with 1 × 10^4^ tachyzoites of T. gondii by IP injection. Open field behavioural testing was carried out 6 days PI. During open field, the (A) number of line crossings, (B) time spent mobile, (C) number of rears and (D) number of defecations during the test were measured. The Y-maze behavioural test was carried out 5 days PI. (E) Percentage alternations and (F) the number of arm entries in the Y-maze were measured. Data analysed by two-way ANOVA with Tukey’s multiple comparisons test, *p < 0.05; **p < 0.01. All data are shown as mean ± SEM. (TIF 133 kb) [file 12974_2017_826_MOESM3_ESM.tif]
